# Every Thursday

**Published:** 1890-05

**Authors:** 


### Every Thursday; An Illustrated Family Journal. Published by Charles Robinson, 150 Fifth Avenue, New York City. Subscription, $2.50 per year; to clergymen, $2.00.

The first number of this neat weekly is before us. It contains a variety of literary articles all very interesting. Among these may be mentioned the following: "William Cullen Bryant's Hymns," "Provincialisms," "Eyes and Ears," "Love as an Embodiment in God," "International Sunday-School Lesson," "White Suns," a story in two parts; "The Oldest Sculpture in the World," etc. There are several very excellent illustrations. W. M. J.

